# Exosomal miRNAs as biomarkers and therapeutic targets in silicosis-related lung fibrosis

**DOI:** 10.1007/s11033-025-10687-w

**Published:** 2025-06-12

**Authors:** Gaurav Gupta, Ahsas Goyal, Baby Ilma, M. M. Rekha, Priya Priyadarshini Nayak, Mandeep Kaur, Anil Khachi, Kavita Goyal, Mohit Rana, A. Rekha, Dennis Chang, Kamal Dua

**Affiliations:** 1 Centre for Research Impact & Outcome, Chitkara College of Pharmacy, Chitkara University, Rajpura, Punjab 140401 India; 2 Centre of Medical and Bio-allied Health Sciences Research, Ajman University, Ajman, United Arab Emirates; 3 Institute of Pharmaceutical Research, GLA University, Mathura, Uttar Pradesh India; 4 Sharda School of Pharmacy, Sharda University, Greater Noida, Uttar Pradesh 201310 India; 5 Department of Chemistry and Biochemistry, School of Sciences, JAIN (Deemed to be University), Bangalore, Karnataka India; 6 Department of Medical Oncology, IMS and SUM Hospital, Siksha ’O’ Anusandhan (Deemed to be University), Bhubaneswar, 751003 Odisha India; 7 Department of Allied Healthcare and Sciences, Vivekananda Global University, Jaipur, Rajasthan, 303012 India; 8 Department of Applied Sciences, Chandigarh Engineering College, Chandigarh Group of Colleges, Jhanjeri, Mohali, Punjab 140307 India; 9 Department of Biotechnology, Graphic Era (Deemed to be University), Clement Town, Dehradun, 248002 India; 10 Uttaranchal Institute of Pharmaceutical Sciences, Uttaranchal University, Dehradun, India; 11 Hospital and Research Centre, Dr. D.Y. Patil Medical College, Pimpri, Pune India; 12 NICM Health Research Institute, Western Sydney University, Westmead, Sydney, NSW 2145 Australia; 13 Graduate School of Health, Faculty of Health, University of Technology Sydney, Sydney, Ultimo, NSW 2007 Australia; 14 Woolcock Institute of Medical Research, Macquarie University, Sydney, NSW 2007 Australia

**Keywords:** Exosomal miRNAs, Silicosis, Lung fibrosis (LF), Biomarkers, Therapeutics, Extracellular vesicles

## Abstract

Silicosis, a form of lung fibrosis (LF), remains a major global health concern because there are few effective ways to diagnose or treat it. Due to their potential function as diagnostic markers and modulators of cancer, exosomal miRNAs are notably featured in more recent advancements. Such exosomal miRNAs offer an invasive approach to diagnosing an illness’s early and accurate nature as they are more stable and measurable in biofluids while illustrating novel cellular changes. Their roles point out this capacity to alter specific molecular processes related to the development of silicosis, such as inflammation, fibrosis, and immunomodulation. Hence, it is possible to find therapeutic targets to decrease fibrosis and diagnostic markers to assess the progression of the disease in these molecules. Recent studies indicate they are involved in fibroblast to myofibroblast transition, macrophage activation in silica, and aberrant signalling such as TGF-β and NF-κB. Delivery methods to employ exosomal miRNAs for therapeutic purposes, including replenishment of beneficial miRNAs and targeted change of detrimental ones, are being developed based on these findings. Nevertheless, when these developments apply to these discoveries in clinical practice, questions about factors such as possible scale, standardisation, and targeting services distribution arise. The diagnostic and treatment possibilities of exosomal miRNAs in LF connected to silicosis are investigated in the present study, with characteristics of revolutionary values and gaps filling the current scale of knowledge in the treatment of silicosis and other similar diseases.

## Introduction

One of the leading causes of pulmonary fibrosis and a global health problem, silicosis is a long-term occupational lung disease caused by prolonged exposure to silica dust, mainly from mining and construction environments. Lung tissue fibrosis, immunological dysregulation, and excessive inflammation are the disease’s hallmarks, which inhibit respiratory capacity and increase the risk of infections and cancers [1, 2]. Dysregulated remodelling of the extracellular matrix, overproduction of pro-inflammatory cytokines, and activation of macrophages induced by silica particles are the principal mediators of fibrotic changes resulting in irreversible alterations. LF, a hallmark of advanced silicosis that fosters pulmonary disease, often leads to considerable morbidity and death. Sensitive biomarkers that can identify disease onset in its early stages are needed, as traditional diagnostic approaches such as imaging and lung biopsies are inadequate for early diagnosis [3, 4]. But there are no cures right now for the condition; the only approved treatments are anti-fibrotic drugs and symptomatic treatment. As we learn more about the molecular mechanisms that underlie fibrosis and silicosis, we stress the importance of exploring new therapeutic targets and treatment strategies. Novel insights into exosomal miRNAs and their roles in cell signaling and fibrosis offer hopeful avenues for early detection and therapy [5, 6]. Relying on modern molecular research, the challenge of silicosis and associated LF should be integrated with clinical practice to foster increased quality of life and clinical outcomes for affected patients. A major occupational health hazard, silicosis-related LF, is associated with high morbidity and mortality, exacerbated by insufficient diagnostic and treatment modalities [7, 8]. There are challenges with early intervention since current approaches primarily utilise imaging and clinical evaluations, which often detect the disease at a later stage. In addition, most treatment modalities are palliative, focusing on symptom control rather than addressing the etiologic drivers of fibrosis. This highlights a fundamental understanding gap in identifying reliable biomarkers for early detection and therapeutic targets for disease modulation [9, 10]. Novel biomarkers with stability, specificity, and the ability to capture dynamic alterations in the status of the pathophysiologic processes in the lung, such as exosomal miRNAs, have the potential to transform practice. Besides functioning as such sensitive diagnostic tools, these molecules also reveal insights into molecular mechanisms in fibrosis [11]. In addition, the progress of molecular medicine and nanotechnology may change the therapeutic landscape for silicosis by creating specific therapeutics. So, scientists can develop personalised medicine approaches to improve early detection, predict disease progression, and help find effective therapies that will mitigate the debilitating manifestations of silica-associated LF [12, 13]. Exosomal miRNAs are an exciting new frontier in diagnosis and therapy that can potentially transform the treatment of complex diseases. These extracellular vesicle (large-order) miRNAs are released from various cells, where miRNAs play a key role in regulating gene expression and modulating intercellular communication and reflect the physiological and pathological states dynamically [14]. Due to their stability in body fluids and the ability of non-invasive liquid biopsy to easily access them, exosomal miRNAs are extremely important biomarkers, which are more sensitive, specific, and simple than conventional diagnostics. Exosomal miRNAs play an important role in disease treatment by targeting key biochemical pathways such as fibrosis, immune response, and inflammation, and they open new avenues for precision medicine [15]. Therapeutically, miRNAs can block harmful miRNAs or introduce beneficial miRNAs in the area of concern to address the fundamental causes of disease formation. Advancements in nanotechnology have furthered their delivery, targeting to maximise efficacy. Hence, exosomal miRNAs could help elucidate disease processes and act as dual-purpose tools for early diagnosis and therapy in silicosis-related LF. Exosomal miRNAs represent a groundbreaking step in personalised and precision medicine by bridging critical gaps in traditional medical approaches and changing the paradigm of therapeutic and diagnostic modalities [16].

Isolation of exosomes by differential ultracentrifugation, size-exclusion chromatography, or precipitation kits often yields heterogeneous vesicle populations contaminated with protein and RNA aggregates, which can bias downstream miRNA profiling [17, 18]. Quantifying exosomal miRNAs using qRT-PCR or next-generation sequencing remains hampered by inconsistent RNA recovery, lack of robust spike-in controls, and absence of universally accepted reference miRNAs, limiting cross-study comparability [19, 20]. Moreover, therapeutic strategies employing miRNA mimics or antagomirs risk unintended off-target gene modulation and immune activation, underscoring the need for stringent specificity validation and safety assessment in preclinical models [21, 22].

This review synthesises current evidence on the roles of six exosomal microRNAs, miR-107, miR-125a-5p, miR-7219-3p, miR-23a-3p, miR-552-3p and let-7i-5p in silicosis-related lung fibrosis. These candidates were chosen for their consistent validation in in vitro and in vivo silicosis models and their mechanistic involvement in key profibrotic and antifibrotic pathways (TGF-β, NF-κB, MAPK, NLRP3). We first describe exosome biogenesis and the selective packaging of these miRNAs following silica exposure; next, we contrast their context-dependent effects on macrophage fibroblast epithelial crosstalk; then, we examine methodological hurdles including exosome isolation purity, miRNA quantification standardization and mitigation of off-target effects; and finally, we highlight translational avenues for leveraging these exosomal miRNAs as non-invasive biomarkers and as vehicles for targeted anti-fibrotic therapy.

### Pathophysiology of silicosis-related lung fibrosis

#### Early inflammation and progressive fibrosis in silicosis

Following inhalation, crystalline SiO₂ particles are phagocytosed by alveolar macrophages, a process that often culminates in “frustrated phagocytosis,” abundant reactive oxygen species (ROS) generation, and secretion of pro-inflammatory cytokines (TNF-α, IL-1β, IL-6) and chemokines that recruit neutrophils and monocytes to the alveolar space [23, 24]​. The resulting acute inflammatory phase is marked by sustained oxidative stress, epithelial injury, and capillary barrier disruption [25, 26]. Chronic, persistent inflammation drives fibroblast recruitment and activation: TGF-β released by both macrophages and injured epithelial cells induces fibroblast-to-myofibroblast differentiation (α-SMA positive), leading to excessive collagen I/III and fibronectin deposition [27, 28]. Concurrently, ROS-mediated cross-linking of matrix proteins and an imbalance between matrix metalloproteinases (MMPs) and tissue inhibitors of metalloproteinases (TIMPs) impair ECM degradation, cementing irreversible matrix accumulation [29, 30]. In addition, chronic activation of NF-κB and the NLRP3 inflammasome perpetuates profibrotic signalling, and epithelial-mesenchymal transition (EMT) further supplements the myofibroblast pool, collectively sustaining the fibrotic milieu [31].

#### Silica exposure, fibrogenesis, and current challenges in diagnosis and therapy

Chronic exposure to silica dust induces a range of cellular and molecular pathways that drive the progression of LF associated with silica dust. Silica particles are inhaled and phagocytosed by macrophages, and ROS and pro-inflammatory cytokines are secreted. This early inflammatory response activates signaling pathways, leading to fibrosis [32]. Key pathways involved in silicosis include the TGF-β signalling pathway, which induces myofibroblast differentiation and fibroblast activity, leading to lung parenchyma remodelling and accumulation of extracellular matrix. In silicosis, it is also known that the exact mechanism of chronic inflammation contributes to fibrogenesis, caused by the persistent activation of immune cells (e.g., lymphocytes, macrophages), resulting in the aggravation of tissue damage [33]. Inflammatory mediators, including TNF-α, IL-1, and IL-6, also potentiate fibrotic response. The deregulation of the above pathways leads to scarification of lung tissue, decreasing respiratory capacity, and increasing the risk of pulmonary hypertension and respiratory failure. The pathogenesis of silicosis has now been better understood, but few diagnostic and treatment methods are available [34]. Existing diagnostic methods primarily depend on imaging techniques such as CT colograms and chest X-rays, which may not always be adequate for accurate disease staging and early detection. There are currently no established therapies to halt or reverse fibrosis progression. Thus, therapeutic management options focus primarily on alleviating the symptoms. Innovative diagnostic biomarkers and targeted therapies are necessary to improve patient outcomes and mitigate the challenges of pulmonary fibrosis due to silicosis [35].

#### Exosomal miRNAs: biogenesis, function, and significance

Exosomal miRNAs play a role in the pathophysiology of lung fibrotic diseases and are vital for intercellular communication. Exosomes are nanosized extracellular vesicles (30–150 nm) released into the extracellular space due to ectodomain shedding and the formation of early endosomes with the budding of the plasma membrane via MVBs [36]. MVBs can then fuse with the plasma membrane to release exosomes into the extracellular milieu. Due to their protective lipid bilayer, exosomes are an ideal delivery vehicle for biomolecules, including miRNAs. miRNAs are selectively incorporated into exosomes through biogenesis, where specific sorting mechanisms that comprise RNA-binding proteins and cellular components recognise miRNA sequences to facilitate their transport. Once inside exosomes, miRNAs are protected from degradation, leading to their constant delivery to far-away target cells [37]. Exosomal miRNAs suppress gene expression by binding to their target mRNAs and inducing translation repression or degradation of target mRNA. This process plays a role in essential cellular mechanisms such as inflammation, fibrosis, and immunological response. Exosome-mediated cell communication is particularly relevant in the context of LF [38]. The signaling mediated by exosomal miRNAs between fibroblasts, macrophages, and epithelial cells participates in the development of fibrosis. Exosome-originating miRNAs regulate key processes, such as TGF-β signalling, critical for fibroblast activation and deposition of ECM components. Therefore, exosomal miRNAs that shift cell-to-cell communication and cellular mechanisms can regulate the progression of pulmonary fibrosis [39].

### Role of Exosomal miRNAs in silicosis therapy and lung fibrosis

Exosomal miRNAs are involved in core pathways regulating inflammation, fibrosis, and tissue repair. They play a crucial role in LF and silicosis therapy. Circulating miRNAs in extracellular vesicles may play a role in these cellular responses and gene expression and may serve as potential therapeutic agents and diagnostic markers [40]. Due to their ability to modulate multiple molecular pathways, exosomal miRNAs present interesting targets for personalised candidates for treating LF connected to silicosis [41]. Regarding the current study, miR-107 is critical to exosomal LF because it modulates significant molecular pathways of fibroblast activation and extracellular matrix deposition. Here, miR-107 inhibits intercellular communication through exosomal transfer, promoting fibrosis progression. MiR-107 can be regarded as a potent biomarker and a therapeutic target for preventing fibrosis in lung-related diseases since its expression is associated with pathological remodelling [42]. Xi et al. also identified that the elevated levels of exosomal miR-107 in the serum of silicosis patients and mice exposed to micron-grade silica particles induce LF. Macrophage-derived exosomes were demonstrated to transfer miR-107 to lung fibroblasts to deregulate their cell cycle pathway and cause CDK6 knockdown with subsequent phenotypic trans-differentiation and fibrosis. Compared with the effects of a positive control inhibitor, the regulatory function of exosomal miR-107 in the pathogenesis of silicosis and the value of exosomes in reducing silica-induced LF were confirmed [43].

**Fig. 1:**
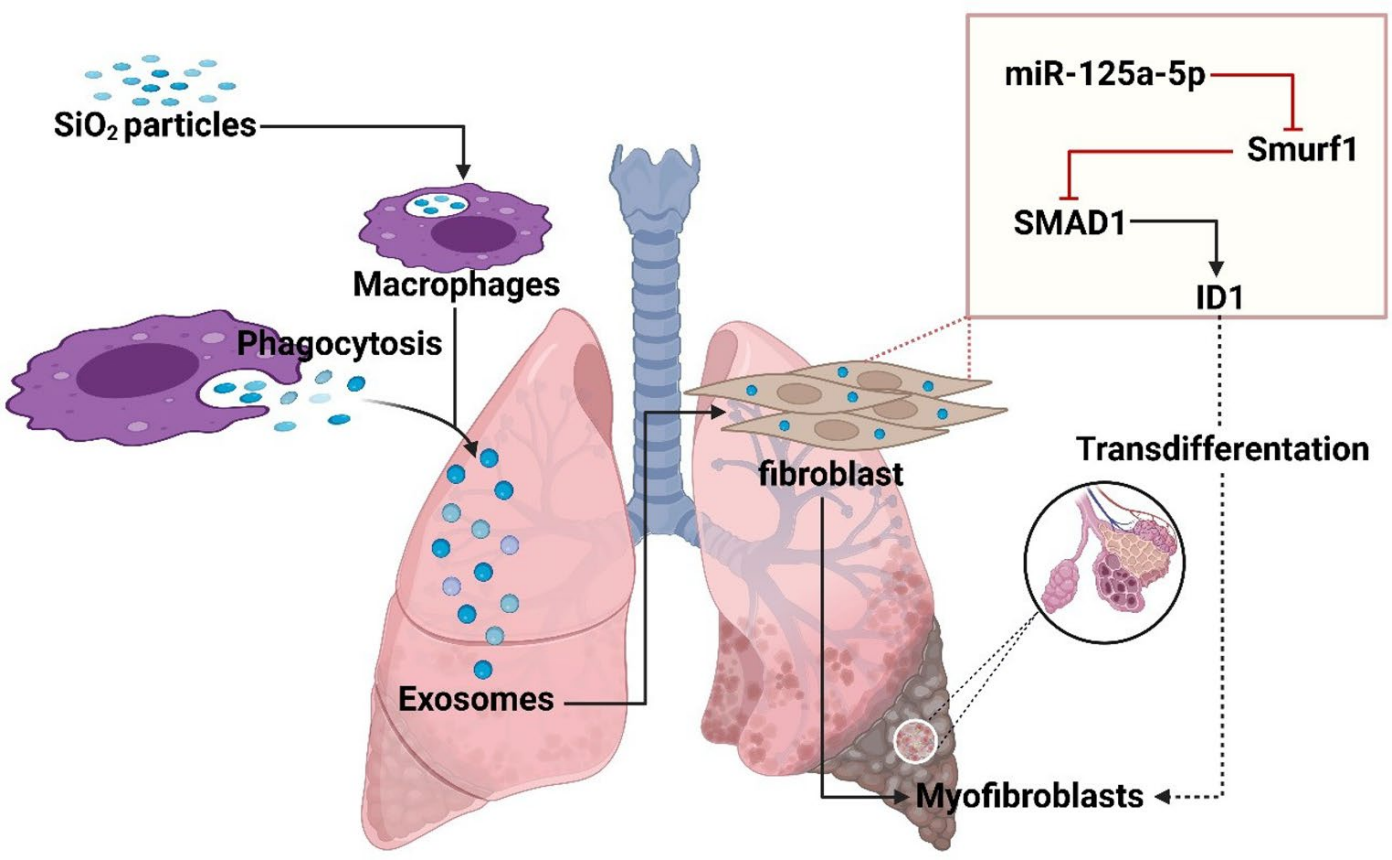
miR-125a-5p–Mediated Myofibroblast Differentiation. This figure describes how SiO₂ particles cause LF. SiO₂ particles cause exosomes carrying fibrotic signals to be released when macrophages phagocytose them. By influencing fibroblast activity, these exosomes promote the development of myofibroblasts. Smurf1 is downregulated by miR-125a-5p, which also increases SMAD1 signaling and ID1 expression two important factors in fibroblast-to-myofibroblast trans-differentiation. This process aids in the deposition of extracellular matrix and the remodeling of fibrotic tissue in LF caused by silicosis. Gaining knowledge of these pathways can help clinicians develop treatments to slow the growth of fibrosis. This fgure was created using Biorender software (Liscence: *DJ28C956RP*)

Macrophage-derived exosomal miR-125a-5p is markedly up-regulated following silica exposure and exerts profibrotic and immunomodulatory effects [44, 45]. Wang et al. used high-throughput sequencing of exosomes from silica-activated macrophages and identified miR-125a-5p as one of the top up-regulated candidates; functional assays then demonstrated that miR-125a-5p directly targets Smurf1 to potentiate TGF-β/Smad signalling upregulating SMAD1, ID1, α-SMA, and collagen and driving fibroblast-to-myofibroblast transdifferentiation in vitro [44]​. Ding et al. extended these findings in a murine silicosis model, showing that systemic administration of a miR-125a-5p antagomir restores Th1/Th2 and Treg/Th17 balance, attenuates NF-κB activation, and reduces collagen deposition and lung fibrosis in vivo [46, 47]. Together, these studies highlight miR-125a-5p’s dual roles: intrinsic activation of fibroblasts and modulation of adaptive immunity as a compelling therapeutic target in silicosis-related lung fibrosis (Fig. 1).

**Fig. 2:**
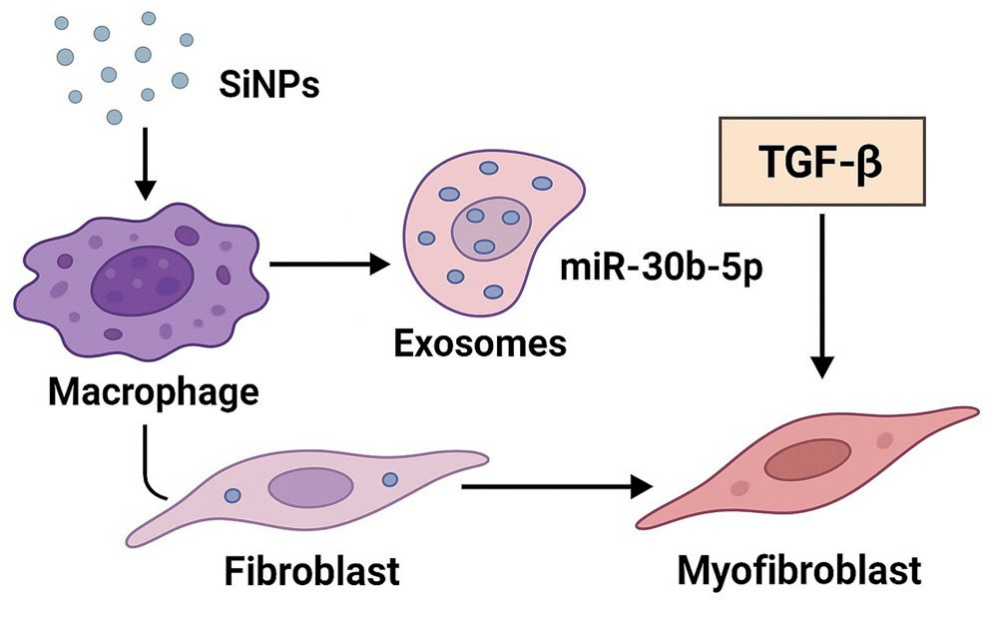
Exosomal miR-30b-5p–Mediated Fibroblast-to-Myofibroblast Transition. Silica nanoparticles (SiNPs) are engulfed by alveolar macrophages, triggering the release of exosomes enriched in miR-30b-5p. Upon uptake by lung fibroblasts, miR-30b-5p activates TGF-β signaling, promoting their differentiation into α-SMA–positive myofibroblasts. This cascade illustrates how exosomal miR-30b-5p links silica exposure to fibrotic remodeling in silicosis. This fgure was created using Microsoft PowerPoint and Smart Servier Medical Art

Silicosis-induced LF has oxidative stress, inflammation, and fibroblast activation, all modulated by MIR-7219-3p. It reduces inflammation responses and extracellular matrix deposition through known genes involved in fibrosis. Silica exposure could mobilise miR-7219-3p in serum as an ideal biomarker for silica-induced LF and associated lung injury and exploitation as a therapeutic approach [48]. Niu et al. examined the role of exosomal miR-7219-3p released by macrophages under silica exposure in FMT in silicosis, and they found that high expression of miR-7219-3p can enhance FMT, cell migration, and proliferation through targeting SPRY1 to activate the RAS/ERK/MAPK pathway. In conclusion, the use of miR-7219-3p as an anti-silicosis therapeutic target was supported by the down-regulation of FMT and the alleviation of silica-fibrosis in vitro and in vivo [49]. Silicosis induces LF mainly through the TGF-β pathway of fibroblast activation, deposition of the extracellular matrix, and epithelial-mesenchymal transition. However, dysregulation worsens lung damage since it prolongs the lung fibrosis and inflammation processes. Targeting TGF-β pathways has therapeutic prognostic value for reducing LF in silicosis or enhancing long-term respiratory disease treatments [50]. In their study, Zhang et al. established that SiNPs induced pyroptosis in macrophages and release of exosomes that induce fibrosis in the lung and fibroblast-myofibroblast transition, using scRNA-sequencing in vitro and in vivo models of silicosis. Exosomal miRNA profiling was demonstrated to alter the TGF-β signaling factors and facilitate fibroblast transdifferentiation. When researchers administered exosomes to the silicotic mice, their fibrosis and inflammation rates were aggravated. These outcomes contrast with the biological functions of exosomal miRNAs in silicosis and several potential treatment methods that focus on exosomes derived from macrophages [51] (Fig. 2).

**Fig. 3:**
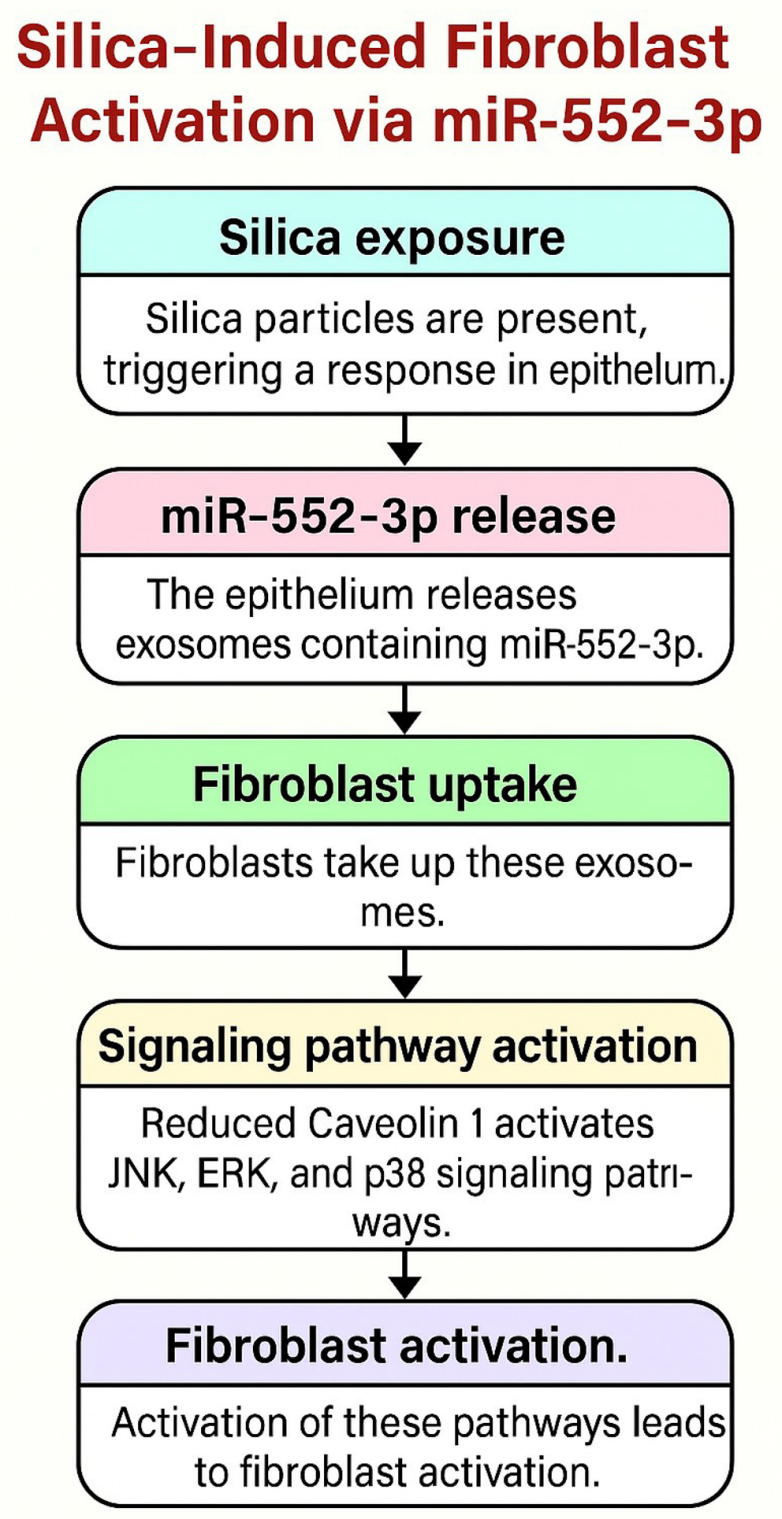
Silica-induced fibroblast activation via miR-552-3p. The figure demonstrates that the regulation of miR-552-3p controls silica-induced fibroblast activation. Silica causes epithelial cells to secrete exosomes with miR-552-3p. The fibroblasts then swallow these exosomes up in a process called phagocytosis. Since miR-552-3p directly targets CAV1 mRNA in fibroblasts, the expression of Caveolin 1 is also reduced. A downregulation of Caveolin 1 constitutively activates the fibroblast, by signaling the JNK, ERK, and p38 pathways. This cascade shows the roles of miR-552-3p in fibroblast activation induced by silica and its possible impact on fibrosis and signaling pathways. This fgure was created using Microsoft PowerPoint

Regulating inflammation, fibrosis, and apoptosis, miR-23a-3p governs critical processes of LF by blocking silicosis. Enhances fibrosis through FACE regulation of fibroblast and matrix facilitation of deposition. MiR-23a-3p holds potential as a targeting treatment and circulating marker for lessening LF and improving lung disease associated with silica-linked results [48]. Chang et al. analysed transcriptome sequencing to investigate the serum exosomal miRNA profile in silicosis patients and determined that serum exosomal miR-23a-3p reduces fibrosis in the lung due to silica. The decreased apoptosis in mice and epithelial cells has been confirmed by employing the miR-23a-3p/CUL3 axis. The role of miR-23a-3p as the diagnostic and therapeutic target for silicosis is likely due to the exosome interchange between macrophages and epithelial cells [52]. Its activation through the NLRP3 inflammasome axis is quoted to cause LF and silicosis besides stimulating cytokines like IL-1β and IL-18 in silica-imparted human mesenchymal stem cells. This leads to fibrotic remodelling and considerable inflammation. By dampening the inflammatory pathways, reducing fibrosis, and enhancing the prognosis of diseases related to silica exposure, NLRP3 has shown novel therapeutic potential [53]. Zhang et al. explored the roles of exosomal circRNA in silica-induced LF and discovered its existence in the serum of the silicosis patient. In this study, they found that through miR-30 b-5p/NLRP3 signalling, this circRNA aggravated macrophage pyroptosis and supported fibroblast activation; overexpressed miR-30 b-5p attenuated pyroptosis and reduced lung inflammation and fibrosis in mice. The findings indicate that circRNA is involved in silicosis and provides data for its early diagnosis and treatment [54]. Caveolin 1 (CAV1) involves many crucial processes in silicosis-induced LF, including fibroblast activation, oxidative stress, and inflammation. Reduces the expression of the molecules that regulate cell health in that it stimulates the accumulation of extracellular matrix and the progression of fibrosis. Parsing CAV1’s role offers insights into its promise as a fibrosis biomarker/therapeutic in lung diseases due to silica [55]. Li et al. found that Exosomal miR-552-3p from BALF of silicosis patients enhances the activation of fibroblasts through targeted inhibition of CAV1. In silica-exposed cells and silicosis models, overexpression of miR-552-3p enriched with CAV1 was reduced, while the fibrotic markers such as fibronectin and α-SMA were escalated. Furthermore, miR-552-3p activates MAPK signaling, which can explain its role in exosomal miRNA-based cross-talk throughout the pathogenesis of silicosis [56] (Fig. 3).

**Fig. 4:**
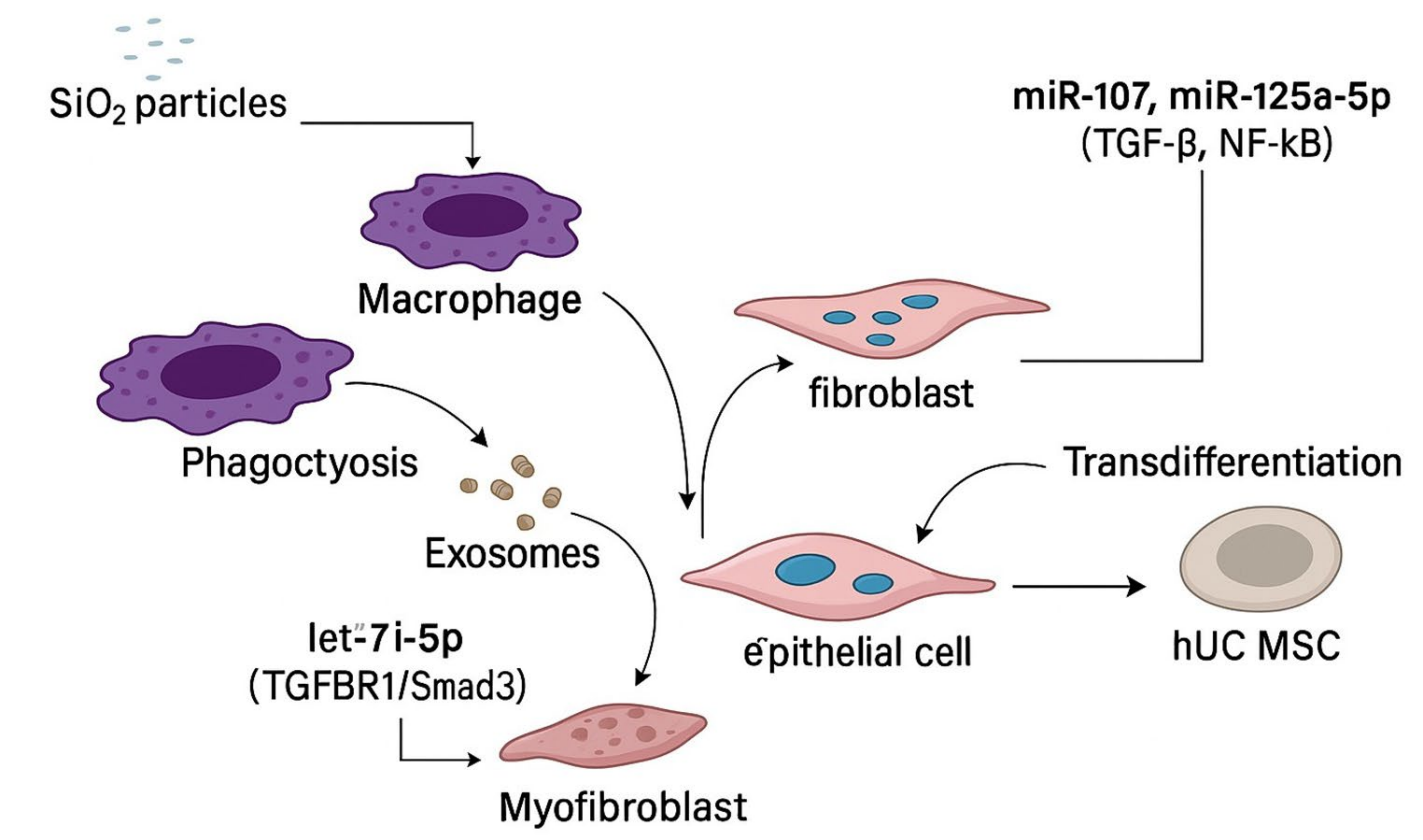
Contextual factors: Key pathways and cellular interactions in Silicosis. This schematic illustrates how inhaled SiO₂ particles are phagocytosed by alveolar macrophages, which in turn release exosomes loaded with key miRNAs. Macrophage-derived miR-107 and miR-125a-5p activate TGF-β and NF-κB signaling in fibroblasts, driving their transdifferentiation into myofibroblasts, while let-7i-5p from hUC-MSCs inhibits TGFBR1/Smad3-mediated fibroblast activation. The diagram also depicts exosomal transfer to epithelial cells, highlighting the multifaceted cellular crosstalk and molecular pathways that underpin silicosis-related lung fibrosis. This fgure was created using Microsoft PowerPoint and Smart Servier Medical Art

LF induced by silicosis depends on TRAF6 activation, which regulates downstream TGF-β and NF-κB signalling [57, 58]. Its activation induces cytokines, fibroblast generation, and extracellular matrix accumulation. It’s possible that the targeting of TRAF6 can prevent these detrimental actions and provide a potential therapeutic strategy for LF and related inflammation precipitated by silicosis [59]. Xu et al. analysed that three-dimensionally cultured hucMSCs released exosomal let-7i-5p that inactivated TGFBR1/Smad3 to inhibit fibroblast activation and ameliorate silica-induced PF. Therefore, using fibroblast and animal model studies proved that hucMSC-derived exosomes transfer let-7i-5p to suppress fibroblast activation. This result shows that hucMSCs exosomes can downregulate silica-induced PF [60]. Silicosis-induced LF depends on the ratio of Th1/Th2 to Treg/Th17 cells. While resulting in inflammation and fibrosis, Th1 cells potentiate fibrosis through fibrogenic mediators released by Th2 cells [61]. The increase and decrease of Treg and Th17 ratios determine the progression of the disease due to increased fibrosis and inflammation. Other possible treatment interventions in silicosis include modifying these cell populations [61]. The study by Ding et al. indicated that Silica-induced macrophages exo-miR125a-5p reprogram fibroblasts by altering the Th1/Th2 and Treg/Th17 ratios. Silicosis patients’ exosomal miR125a-5p, TGF-β1, IL-17 A, and IL-4 blood levels were elevated and related to silicosis progression [46]. Both T cells, exosomes, and macrophages increased the expression of miR125a-5p in vitro, which led to fibroblast-myofibroblast transdifferentiation and inhibition of T cell development [62]. Because of these findings, exosomal miR125a-5p could be potentially used as a candidate target for the therapy of silicosis (Fig. 4; Table 1).

**Table 1:** Exosomal miRNAs in silicosis-related lung fibrosis

Exosomal miRNA	Key Findings	Model Systems	Limitations	Reference
miR-107	Macrophage-derived exosomal miR-107 knocks down CDK6 in lung fibroblasts, driving proliferation and ECM (collagen/fibronectin) deposition via TGF-β/NF-κB signalling.	Patient serum exosomes; silica-exposed mice; primary murine lung fibroblasts	No validation in human lung tissue; rodent-only assays; limited mechanistic depth.	[43]
miR-125a-5p	Targets Smurf1 to amplify TGF-β/Smad signalling and TRAF6 to modulate NF-κB/Th1-Th2 balance, driving fibroblast-to-myofibroblast transition and immune reprogramming.	In vitro: exosomes from silica-activated macrophages + primary fibroblasts; in vivo: murine silicosis model with miR-125a-5p antagomir	Long-term immune safety uncharacterized; lacks clinical patient data.	[44, 46, 63]
miR-30b-5p	Suppresses NLRP3-mediated pyroptosis in macrophages and attenuates fibroblast activation, reducing lung inflammation and fibrosis.	Serum exosomes from silicosis patients; macrophage/epithelial co-cultures; mouse silicosis model	Dose–response effects on pyroptosis vs. repair undefined; no direct human correlation.	[54]
miR-552-3p	Downregulates Caveolin-1, activates MAPK, and upregulates fibronectin/α-SMA to promote fibroblast activation and matrix deposition.	BALF exosomes from patients; in vitro human fibroblasts; rabbit silicosis model	Cell-type specificity not tested; clinical severity correlation absent.	[58]
let-7i-5p	Targets TGFBR1/Smad3 to inhibit fibroblast activation and ameliorate silica-induced pulmonary fibrosis.	3D-cultured hUC-MSC exosomes; in vitro human fibroblasts; mouse silicosis model	Biodistribution and immunogenicity of MSC exosomes not fully assessed; long-term efficacy unknown.	[60]
miR-23a-3p	Modulates epithelial apoptosis via CUL3/NLRP3 axis, protecting against silica-induced cell death and subsequent fibrosis.	Serum exosomes from silicosis patients; murine and human epithelial lines; mouse fibrosis model	Potential off-target inflammasome effects; human safety and validation data lacking.	[52]

### Context-dependent activities of exosomal miRNAs in silicosis

The fibrotic or antifibrotic outcome of a given exosomal miRNA hinges on multiple variables: the cell of origin (e.g., activated macrophages versus mesenchymal stem cells), the recipient cell type (fibroblast, epithelial or immune cell), disease stage and the miRNA dose delivered [64]. For example, macrophage-derived miR-125a-5p drives fibroblast trans-differentiation via TGF-β signalling in vitro but can reprogram T cell subsets to attenuate inflammation in antagomir-treated mice [65]​​​. Likewise, miR-23a-3p reduces epithelial apoptosis at low doses through the CUL3/NLRP3 axis, whereas supraphysiological levels may impair epithelial repair ​ [66]. Modulators such as exosome surface ligands, co-packaged proteins, and the surrounding cytokine milieu further dictate miRNA uptake and downstream signaling [67]. Recognising these nuances underscores the importance of precise dosing, cell-specific targeting strategies, and rigorous in vivo validation under physiologic conditions.

### Exosomal miRNAs as biomarkers in silicosis

Exosomal miRNAs afford promising novel biomarkers in silicosis with potential diagnostic importance, attributed to their unique characteristics and involvement in disease progression. Various cell types, including fibroblasts and macrophages, release these tiny non-coding RNAs in exosomes in response to silica exposure [68]. Specific miRNAs (such as miR-125a-5p, miR-107, and miR-30 b-5p) present in the exosomes of silicosis patients have been shown to correlate significantly with silicosis severity and progression. Their expression levels, easily measurable in biological fluids such as serum, bronchoalveolar lavage fluid (BALF), and plasma, mirror the stage of fibrosis, inflammation, and immunological dysregulation characteristic of silicosis [69]. Exosomal miRNAs are reliable early diagnostic and prognostic biomarkers because they can analyse disease progression and closely correlate with clinical endpoints such as fibrosis markers and lung function decline. Exosomal miRNAs exhibit diverse advantages as potential biomarkers. They comprise exceptional specificity resulting from their tight and systematic expression patterns, remarkable exosome stability by imprisonment from enzymatic degradation, and non-invasiveness using liquid biopsy [70]. Exosomal miRNAs are better than conventional protein-based indicators in terms of these properties. They bridge the divide between clinical diagnostics and the practice of specific medicine by enabling the investigation of the dynamic cellular and molecular changes that grant a higher-resolution elucidation of the pathophysiology of silicosis [71]. With advancing research, early identification, treatment response analysis, and personalised therapy for each patient can be made possible in silicosis diagnostics through an exosomal approach to miRNA profiling. Therefore, exosomal miRNAs provide a novel strategy for modulating silicosis-related LF [72].

### Therapeutic potential of exosomal miRNAs in silicosis

Exosomal miRNAs play a role in key pathogenic processes and influence fibrosis. The therapeutic potential of exosomal miRNAs in silicosis is substantial. These exosomal miRNAs target fibroblasts, immune cells, and inflammatory pathways and act as endogenous mediators of intercellular communication [73]. miRNAs, such as miR-125a-5p and miR-107, have been shown to regulate fibroblast trans-differentiation and extracellular matrix deposition by modifying signalling pathways such as TGF-β and NF-κB. MiRNA, such as let-7i-5p, released from MSC-derived exosomes, can prevent fibroblast activation to reduce silica-induced fibrosis. Novel methods are required to administer these miRNAs therapeutically, including formulation of exosomes for delivery concentrated to the lungs and alteration of the miRNA payload through synthetic changes or miRNA mimics [74]. Surface functionalization and aerosolised delivery methods improve exosome stability, bio-distribution, and absorption efficiency. In preclinical studies using animal models of silicosis, exosome-mediated miRNA therapies have been effective in reducing pulmonary inflammation, inhibiting fibroblast activation, and reversing fibrosis. Despite their infancy, medical studies suggest that these findings could one day be translated into therapeutic strategies [75]. Engineered exosomes with miRNA cargo have emerged as a newer strategy to prevent fibrosis. They offer several advantages, including their ability to demonstrate efficient cell-specific targeting and low immunogenicity. In addition, the ability of exosomes to overcome biological barriers makes them a versatile carrier for miRNA delivery. These advancements underscore the potential of exosomal miRNAs as a game-changing therapeutic option for silicosis, paving the way for personalised, non-invasive therapies aimed at preventing or repairing LF [76, 77].

Although exosomal miRNAs offer a biocompatible, low-immunogenicity platform with an innate ability to cross biological barriers, their translation into the clinic is hampered by several technical and regulatory hurdles: high‐purity isolation and scalable production under GMP conditions, efficient and reproducible miRNA loading/enrichment, precise tissue‐ or cell‐type targeting to avoid off‐target effects, and comprehensive safety profiling to rule out undesired immunogenicity or toxicity [78, 79]. At the same time, advances in exosome engineering, such as surface ligand conjugation for homing, hybridisation with synthetic nanovesicles for enhanced stability, and bioreactor‐based culture systems for large‐scale manufacturing are rapidly maturing [80]. Together, these developments set the stage for exosomal miRNAs to become precision delivery vehicles that can be personalised to individual patients’ disease phenotypes, combined with gene‐editing tools or anti‐fibrotic drugs, and rigorously controlled for dose, purity, and safety in future preclinical and clinical studies.

### Current challenges, limitations, and future directions

While individual studies underscore the promise of exosomal miRNAs such as miR-107, miR-125a-5p, and let-7i-5p in modulating fibrotic and inflammatory pathways, a holistic appraisal reveals several critical gaps [81]. First, nearly all data is derived from rodent models or ex vivo human cells, with little direct clinical validation in silicosis patients. Second, methodological inconsistencies ranging from exosome isolation protocols to miRNA quantification, opaque inter-study comparisons, and hinder meta-analysis. Third, the impact of patient variables (e.g., disease stage, comorbidities, genetic background) on exosomal cargo remains unexplored, as does engineered exosomes’ long-term safety and biodistribution. Future research must prioritize standardized isolation and reporting guidelines, integrate multi-omics profiling across diverse patient cohorts, and conduct rigorous dose-finding and toxicology studies in large-animal and early-phase human trials to advance toward clinical translation. Moreover, combining exosomal miRNAs with complementary anti-fibrotic agents or gene-editing approaches may yield synergistic benefits, ushering in truly personalised therapies for silicosis-related lung fibrosis.

There are still challenges limiting silicosis-related LF exosomal miRNAs from global clinical translation. Isolation and characterisation of exosomes is a considerable technological hurdle due to their heterogeneity and diversity of biological sources. Often used methods, such as size-exclusion chromatography or ultracentrifugation, create low levels of purity, which might affect the quality and accuracy of miRNA profiles on exosomes [82, 83]. Exosomal miRNAs must also be exquisitely characterised by cutting-edge and standardised methods, such as quantitative PCR or next-generation sequencing, that are still being adjusted for repeatability. Another challenge is the selective distribution of exosomal miRNAs to specific tissues, such as lung fibroblasts [84]. Although exosomes are naturally used to transport biomolecules, no established exosome modification techniques would allow us to coat or alter their surface characteristics for targeting specific tissues, making it difficult to enhance their preferential absorption and targeting properties. Thus, exosome-based therapies also face scalability and safety problems [85]. Exosomes are widely considered biocompatible, but more investigation is necessary to elucidate their potential immunogenic, toxic, and longer-term effects on organ systems. Moreover, exercising greater exosome production for medical use is required and demands reproducible, cost-effective methods to maintain their activity and integrity. For LF in silicosis being helped by these nano-agents, improving these technological, delivery, and safety challenges is critical to realising the full potential of exosomal miRNAs as therapeutic agents and diagnostic indicators [86].

## Conclusion

In this review, we have synthesised evidence that six exosomal miRNAs, including miR-107, miR-125a-5p, miR-7219-3p, miR-23a-3p, miR-552-3p, and let-7i-5p, critically regulate silicosis-related lung fibrosis by modulating key profibrotic and inflammatory pathways (TGF-β, NF-κB, MAPK, NLRP3). Their remarkable serum and bronchoalveolar lavage fluid stability underpins their promise as non-invasive biomarkers for early diagnosis, disease staging, and therapeutic efficacy monitoring. Furthermore, engineered exosomes loaded with anti-fibrotic miRNAs constitute a novel delivery platform that selectively targets lung fibroblasts with minimal immunogenicity. While challenges remain in standardising exosome isolation, precise tissue targeting, scalable production and rigorous safety validation, exosome engineering and biomanufacturing advances bring us closer to clinical translation. Moving forward, harmonised protocols, large-scale clinical validation, and integration with existing antifibrotic therapies will be essential to realise exosomal miRNAs’ full diagnostic and therapeutic potential in silicosis-associated lung fibrosis.

## Data Availability

No datasets were generated or analysed during the current study.
